# Drug Repurposing in Glioblastoma Using a Machine Learning-Based Hybrid Feature Selection Approach

**DOI:** 10.3390/ijms262411871

**Published:** 2025-12-09

**Authors:** Erdal Tasci, Kevin Camphausen, Andra Valentina Krauze

**Affiliations:** Radiation Oncology Branch, Center for Cancer Research, National Cancer Institute, National Institutes of Health, 9000 Rockville Pike, Building 10, CRC, Bethesda, MD 20892, USA; erdal.tasci@nih.gov (E.T.); camphauk@mail.nih.gov (K.C.)

**Keywords:** glioblastoma, drug sensitivity, cell line, feature selection, machine learning, pattern recognition

## Abstract

Glioblastoma (GBM) is a fatal and aggressive form of brain cancer, described by rapid progression, poor prognosis, and limited treatment options. This study aims to apply a hybrid of two popular feature selection methods for the categorization of drug sensitivity features in GBM versus other cancer cell lines by employing a rank-based weighting combination scheme to identify discriminative drug compound feature sets. This approach is necessary to reduce dimensionality and enhance classification performance while increasing the interpretability of the prediction model. The experimental results indicate that the utilized machine learning (ML)-driven feature selection approach achieves more than 95% accuracy value and obtains less than or equal to 11 selected features for each drug sensitivity metric on Genomics of Drug Sensitivity in Cancer (GDSC) datasets with high-dimensional space. Our drug compound-based findings demonstrate that our feature selection approach improves model stability and performance, paving the way for more precise and clinically actionable advancements in GBM research.

## 1. Introduction

Glioblastoma (GBM) is the most aggressive and devastating primary cancer of the central nervous system, with a poor prognosis and limited treatment options [1]. Globally, brain and other CNS cancers are responsible for around 330,000 incident cases and 227,000 deaths per year [2]. GBM is the most commonly occurring malignant brain tumor in adults, representing 14.6% of all brain and CNS tumors and 48.3% of malignant tumors in US registry data [3]. The population-based incidence of GBM in the US is 3.19 cases per 100,000 people, with a median age of 64 years and higher rates in males [4]. A systematic review in the US shows that GBM patients need to pay very expensive direct costs (i.e., USD 400–430 k per patient [5]). For newly diagnosed GBM, the standard treatment approach involves maximal surgical resection, followed by concurrent chemotherapy (CRT), followed by adjuvant temozolomide (TMZ) [6,7,8,9]. On average, patients with a diagnosis of GBM survive 14 to 15 months following their diagnosis [10]. Although significant progress has been made in molecular classification and proteogenomic characterization [11,12,13,14,15], novel approaches [16,17] have not resulted in improved outcomes, and standard of care (SOC) management has remained unchanged in over 20 years [6]. GBM tumors frequently recur secondary to tumor resistance [18]. Several critical hallmarks of cancer pathways have been implicated in driving GBM resistance with crossover into all modalities of cell kill, including radiation and chemotherapy that comprise the base of SOC [19,20]. Drug resistance in GBM has remained a critical barrier, and utilization of existing therapeutic agents with transferability to GBM has been limited by both target specificity and blood–brain barrier penetration limitations. Given ongoing pressure to optimize management while maximizing speed, time, cost, and accuracy, there is increased focus on drug repurposing, particularly to leverage drugs that are already Food and Drug Administration (FDA) approved for use in humans [21,22,23]. There are currently no biomarkers for GBM to direct management based on resistance patterns, and GBMs are highly heterogeneous tumors [24]. To identify potential drug sensitivity in GBM, cell lines, gene expression profiles, and PDX models have been utilized to probe the possibility of response [25,26]. Modern nanoscale imaging, particularly using atomic force microscopy and other scanning-probe methods, can also provide more than just high-resolution structural snapshots: they enable quantitative, single-cell measurement of mechanical and biophysical parameters such as stiffness and adhesion [27]. This means that atomic force microscopy-based nanomechanical profiling can reveal biophysical “signatures” distinguishing healthy glial cells from GBM cells. Different GBM cell lines can also show significant differences in nanomechanical and viscoelastic properties, cytoskeletal organization, and migration behavior [28]. These properties can serve as a nanomechanical signature or biomarkers of GBM cell heterogeneity, for aggressiveness, and potentially disease progression [28]. While several datasets can be employed for drug sensitivity work, consistent labels and standardized dose response curves, as well as strong connections to matched genomic, target, and pathways assignment, are key to arrive at clinically actionable results [29]. Drug sensitivity datasets are, as a result, composed of a large number of variables (i.e., features), including several that can potentially define the sensitivity to a drug, hence carry wide dimensionality which varies across datasets. In addition, datasets can have significant amounts of missing data. Feature selection is a critical process for dimensionality reduction to enhance data visualization and interpretation, minimize computational and storage requirements, accelerate learning model training processes, and overcome the curse of dimensionality to boost predictive model performance [30,31]. With the development and advances in technology and artificial intelligence (AI)-based methods, such as machine learning models, feature selection can be carried out efficiently in complex biomedical datasets wherein patient or cell line management is varied to optimize speed, time, cost, and accuracy. By employing robust evaluation metrics and leveraging filter, wrapper, or embedded techniques, the selection of specific signals related to defined class labels, e.g., drug compounds in patients with GBM, can result in robust biomarker candidates. In this study, we introduce a fused machine learning-based feature selection and weighting method that finds significant drug compound candidates by discriminating GBM signals from other cancer cell lines for Genomics of Drug Sensitivity in Cancer (GDSC) datasets.

The key contributions of this research, categorized into technical and clinical aspects, are summarized as follows:

**Technical aspects**

- To the best of our knowledge, this is the first study that employs a combined feature selection and weighting methodology to select drug compounds by discriminating GBM cell lines versus other cell lines by employing four drug sensitivity metrics separately, namely, LN_IC50 (Half Maximal Inhibitory Concentration), AUC (Area Under the Curve), RMSE (Root Mean Squared Error), and Z_SCORE (Standard score).
- We assessed the effects of varying feature selection and weighting task weights on the classification model’s performance.
- To improve the impact of reliability and fairness of performance estimates under imbalanced class distribution in the GDSC1 and GDSC2 datasets, we employed stratified five-fold cross-validation, maintaining the original class proportions within each data fold.
- We conducted comprehensive experiments for the evaluation of feature selection and weighting across the ensemble machine learning classification model, namely, Random Forest (RF), to identify the optimal drug compound set among all combination sets and the minimal feature subset necessary for precise classification.
- Feature weighting is a crucial stage in determining which features will be used at the final decision point among the various feature names that emerge during the cross-validation process, and in addressing this problem.
- To observe the impact of the selected feature set of GBM cell lines on model prediction performance for each dataset and drug sensitivity metric, we plotted SHAP (SHapley Additive exPlanations)-based plots.

**Clinical aspects**

- We leveraged GDSC datasets, employing feature selection techniques to identify signals that differentiate GBM cell lines vs. other cell lines. This is a novel finding, as drug compound-based GBM cell line profiles with machine learning (ML)-based methodology have not been previously characterized.
- We discussed the potential interpretation of the identified critical features in association with GBM.

The rest of this paper is organized as follows: Section 2 presents a comprehensive overview of the experimental process, performance metrics, and computational results. Section 3 discusses the findings and their implications for drug repurposing. Section 4 describes the dataset employed for feature selection and weighting methodology. Lastly, Section 5 summarizes the conclusions of our research and explores potential avenues for future research.

## 2. Results

In this section, the experimental process and the performance metric employed for feature selection and classification are described. Then, the comprehensive computational results are provided according to each different combination of the scheme, datasets, and metrics.

### 2.1. Experimental Process

**Figure 1:**
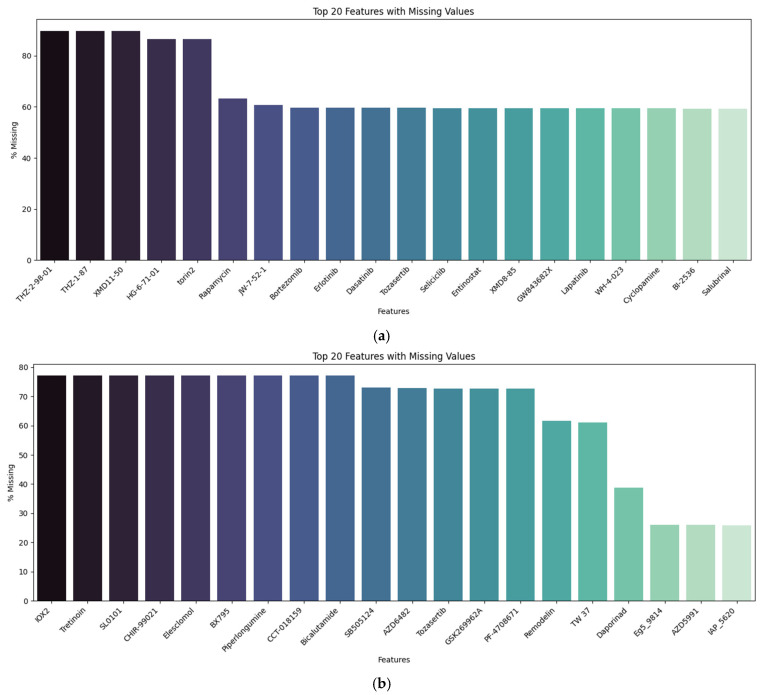
Missing value example statistics for the GDSC1 and GDSC2 datasets based on the AUC metric (**a**) GDSC1 and (**b**) GDSC2 dataset.

To perform the proposed methodology, we employed Python’s (version 3.9) scikit-learn library (version 1.6.1) for implementing the machine learning model and the mRMR (Minimum Redundancy–Maximum Relevance) package version 0.2.8 for the feature selection process. For SHAP analysis, we also utilized the SHAP package version 0.48.0. All experiments were conducted on a macOS Sequoia 15.6.1 MacBook Pro, equipped with a 16-core Apple M3 Max processor and 128 GB of LPDDR5 memory. Feature selection and weighting methodology are employed to identify the compounds that discriminate GBM cell lines, then the GDSC datasets are transformed into a pivot table organized by cell line and cancers (i.e., GBM vs. others) with all potential drug compounds for each drug sensitivity metric. We used the mean aggregation function for this process. Then, we applied the iterative imputation method [32] for the missing data imputation process for each metric and dataset. As all features include missing values in both datasets, we utilized the multivariate and powerful missing data imputation approach that estimates each feature value from all the others. This is why we do not remove missing feature values in this study. We chose full dataset imputation to maintain consistency across large-scale experiments and avoid fold-specific variability, and provide computational efficiency for this study. Although this approach may introduce limited information leakage, it maintains robustness within the biomedical setting. Each feature showed a varying level of missingness. The pattern appeared largely random, with no evident links to specific sample features. For example, Figure 1 illustrates the missing value statistics for the GDSC1 and GDSC2 datasets, based on the AUC metric, the top 20 related missing features, and the corresponding missing ratios.

To ensure optimal results, we employed one of the most effective machine learning models, Random Forest (RF). The predictive model was utilized in both the feature weighting-based selection and classification stages. In this study, we applied stratified five-fold cross-validation to obtain performance results, provide consistent data, and improve representation by reducing the bias in training and evaluation. To preserve uniformity in dataset results, we fixed the random state at 0 for the Random Forest model.

### 2.2. Performance Metrics

To evaluate the effectiveness of our utilized hybrid feature selection and weighting method on drug compound-based cancer cell line datasets, we considered the classification accuracy rate in this research. This metric serves as a direct indicator of the predictive or discriminative ability of the feature selection process to correctly categorize samples into GBM or other cancer cell lines.

Classification accuracy (ACC) is defined as the ratio of correctly classified samples to the total dataset samples. In other words, accuracy is computed by adding the number of true positive and true negative predictions and then dividing this sum by the total number of true positives, false negatives, true negatives, and false positives [33], as defined in Equation (1).

(1) $$ACC=\frac{\mathrm{TP}+\mathrm{TN}}{\mathrm{TP}+\mathrm{TN}+\mathrm{FP}+\mathrm{FN}}$$

where TP, FN, TN, and FP represent the number of true positives, false negatives, true negatives, and false positives, respectively. We aimed to find the minimum number of selected features with the highest accuracy rate among all combination schemes, including rank-based feature weights for each dataset and drug sensitivity metric.

### 2.3. Computational Results

This subsection presents the effect of our feature selection and weighting approach on the performance of the machine learning model employed (i.e., RF) for different drug sensitivity metrics-based GDSC datasets.

#### 2.3.1. The Effects of Feature Selection and Weighting Method on Classification Model Performance for the Categorization of GBM Cell Lines Versus Other Cell Lines on GDSC Datasets

We assessed the performance of LASSO (Least Absolute Shrinkage and Selection Operator) and mRMR-based feature selection methods using rank-based weighting schemes (with weights of 1 and 2) using stratified five-fold cross-validation. The computational results of these experiments, detailing the weight count (‘k’), and the number (#) of selected features, are fully tabulated in Table 1, Table 2, Table 3, Table 4, Table 5, Table 6, Table 7 and Table 8. The changes in color, from red to green, in the tables represent the lowest (red) accuracy rate values to the highest accuracy rate values (green).

#### 2.3.2. GDSC1 Dataset Results

The first analysis used the area under the curve (AUC)-based drug data from the GDSC1 dataset, the highest (best) accuracy value with the minimum number of selected features is achieved by choosing 10 features, assigning LASSO = 1 and mRMR = 2 rank-based weights, a total weight value of 6, Random Forest (RF) model with a 96.592% accuracy rate (ACC) (Table 1). In this study, we selected the RF model as it provides more efficient results than other prediction models. If we keep the total weight value around the highest, the number of selected features will be the lowest (e.g., selected features will be most discriminative and informative; however, some information loss can be possible in this case), as this operation chooses only features selected by both feature selection methods for each fold of the cross-validation.

**Table 1:** The impact of the feature selection and weighting method in terms of the accuracy rate (%) for the AUC-based GDSC1 dataset.

LASSO = 1 and mRMR = 2			LASSO = 2 and mRMR = 1		
k	# of Features	RF	k	# of Features	RF
1	132	96.592	1	132	96.592
2	91	96.592	2	120	96.592
3	68	96.592	3	78	96.592
4	52	96.592	4	74	96.592
5	36	96.592	5	63	96.592
6	**10**	**96.592**	6	60	96.592
7	6	96.591	7	45	96.592
8	3	96.488	8	44	96.592
9	3	96.488	9	31	96.592
10	3	96.488	10	30	96.592
11	3	96.488	11	5	96.487
12	2	96.488	12	3	96.488
13	2	96.488	13	3	96.488
14	1	94.319	14	2	96.488

The second analysis used the half maximal inhibitory concentration-based (i.e., LN_IC50) drug data from the GDSC1 dataset; the highest (best) accuracy value with the minimum number of selected features is achieved by choosing 11 features, assigning LASSO = 2 and mRMR = 1 rank-based weights, a total weight value of 11, with a 96.798% ACC (Table 2). Half maximal inhibitory concentration represents the concentration of a drug needed to inhibit 50% of the biological activity. Greater drug potency leads to lower IC50 values [34]. We can say that ACC values are generally equal to or higher than 96.592% due to the dataset imbalance characteristics. Furthermore, if we use only one most significant and discriminative feature (i.e., drug compound), it will provide a 93.594% ACC value with the RF model. In addition to using only one feature, when only two features are used, our feature selection and weighting methodology provide a 96.180% ACC value (Table 2).

**Table 2:** The impact of the feature selection and weighting method in terms of the accuracy rate (%) for the LN_IC50-based GDSC1 dataset.

LASSO = 1 and mRMR = 2			LASSO = 2 and mRMR = 1		
k	# of Features	RF	k	# of Features	RF
1	155	96.592	1	155	96.592
2	94	96.592	2	151	96.592
3	71	96.592	3	90	96.592
4	54	96.592	4	90	96.592
5	38	96.592	5	71	96.592
6	15	96.798	6	68	96.592
7	14	96.695	7	51	96.592
8	10	96.488	8	49	96.592
9	7	96.591	9	36	96.695
10	6	96.591	10	35	96.592
11	5	96.488	11	**11**	**96.798**
12	3	96.385	12	5	96.385
13	3	96.385	13	4	96.488
14	1	93.594	14	2	96.180

For the Root Mean Squared Error-based (i.e., RMSE) drug compounds regarding the GDSC1 dataset, the highest (best) accuracy value with the minimum number of selected features is obtained with 3 selected features by assigning LASSO = 1 and mRMR = 2, or LASSO = 2 and mRMR = 1 with a 96.798% ACC using the RF model (Table 3). If the number of selected features is more than one, this methodology obtains more than a 96% ACC for this type of dataset.

**Table 3:** The impact of the feature selection and weighting method in terms of the accuracy rate (%) for the RMSE-based GDSC1 dataset.

LASSO = 1 and mRMR = 2			LASSO = 2 and mRMR = 1		
k	# of Features	RF	k	# of Features	RF
1	141	96.592	1	141	96.592
2	95	96.592	2	136	96.592
3	68	96.592	3	90	96.592
4	47	96.592	4	87	96.592
5	32	96.592	5	65	96.592
6	16	96.695	6	64	96.592
7	14	96.695	7	46	96.592
8	11	96.592	8	46	96.592
9	11	96.592	9	32	96.592
10	5	96.385	10	30	96.592
11	4	96.488	11	14	96.592
12	**3**	**96.798**	12	10	96.695
13	**3**	**96.798**	13	4	96.488
14	1	93.906	14	**3**	**96.798**

For the Z_SCORE-based drug compounds regarding the GDSC1 dataset, the highest (best) accuracy value with the minimum number of selected features is obtained with 9 selected features, assigning LASSO = 1 and mRMR = 2 rank-based weights, a total weight value of 9, and a 96.799% ACC (Table 4).

**Table 4:** The impact of the feature selection and weighting method in terms of the accuracy rate (%) for the Z_SCORE-based GDSC1 dataset.

LASSO = 1 and mRMR = 2			LASSO = 2 and mRMR = 1		
k	# of Features	RF	k	# of Features	RF
1	178	96.592	1	178	96.592
2	120	96.592	2	175	96.592
3	93	96.592	3	117	96.592
4	61	96.592	4	114	96.592
5	39	96.695	5	90	96.592
6	14	96.695	6	88	96.592
7	13	96.592	7	58	96.592
8	11	96.488	8	56	96.592
9	**9**	**96.799**	9	36	96.695
10	4	96.696	10	35	96.695
11	4	96.696	11	10	96.798
12	3	96.179	12	8	96.695
13	3	96.179	13	4	96.696
14	1	94.111	14	3	96.179

#### 2.3.3. GDSC2 Dataset Results

When the GDSC2 dataset is used with our feature selection and weighting method, the computational results are shown in Table 5, Table 6, Table 7 and Table 8. For the AUC-based drug compounds regarding the GDSC2 dataset, the highest (best) accuracy value with the minimum number of selected features is achieved by choosing 6 features, assigning LASSO = 1 and mRMR = 2 rank-based weights, a total weight value of 7, Random Forest (RF) model with a 96.573% ACC (Table 5). All results for this drug sensitivity metric and dataset are higher than a 96% ACC.

**Table 5:** The impact of the feature selection and weighting method in terms of the accuracy rate (%) for the AUC-based GDSC2 dataset.

LASSO = 1 and mRMR = 2			LASSO = 2 and mRMR = 1		
k	# of Features	RF	k	# of Features	RF
1	76	96.470	1	76	96.470
2	51	96.470	2	70	96.470
3	32	96.470	3	42	96.470
4	23	96.470	4	40	96.470
5	15	96.262	5	26	96.470
6	7	96.365	6	25	96.470
7	**6**	**96.573**	7	17	96.470
8	5	96.159	8	14	96.470
9	5	96.159	9	9	96.366
10	5	96.159	10	9	96.366
11	4	96.055	11	5	96.159
12	4	96.055	12	4	96.055
13	4	96.055	13	4	96.055
14	2	96.262	14	4	96.055

For the half maximal inhibitory concentration-based drug compounds regarding the GDSC2 dataset, the highest accuracy value with the minimum number of selected features is achieved by choosing 9 features, assigning LASSO = 2 and mRMR = 1 rank-based weights, a total weight value of 11, with a 96.677% ACC (Table 6). We can say that all results are around 96% for this specification.

**Table 6:** The impact of the feature selection and weighting method in terms of the accuracy rate (%) for the LN_IC50-based GDSC2 dataset.

LASSO = 1 and mRMR = 2			LASSO = 2 and mRMR = 1		
k	# of Features	RF	k	# of Features	RF
1	80	96.470	1	80	96.470
2	49	96.573	2	79	96.470
3	37	96.470	3	48	96.470
4	28	96.574	4	46	96.573
5	22	96.470	5	35	96.574
6	12	96.677	6	33	96.574
7	12	96.677	7	26	96.574
8	10	96.365	8	24	96.574
9	8	96.573	9	20	96.574
10	7	96.573	10	20	96.574
11	6	96.469	11	**9**	**96.677**
12	4	96.468	12	6	96.470
13	4	96.468	13	5	96.262
14	4	96.468	14	3	96.366

For the RMSE-based drug compounds regarding the GDSC2 dataset, the highest accuracy value with the minimum number of selected features is obtained with 8 selected features by assigning LASSO = 2 and mRMR = 1, with a total weight value of 12, and a 96.470% ACC using the RF model (Table 7). Although there are many alternatives in terms of the best ACC value, we chose this one due to the minimum number of features selected.

**Table 7:** The impact of the feature selection and weighting method in terms of the accuracy rate (%) for the RMSE-based GDSC2 dataset.

LASSO = 1 and mRMR = 2			LASSO = 2 and mRMR = 1		
k	# of Features	RF	k	# of Features	RF
1	92	96.470	1	92	96.470
2	61	96.470	2	87	96.470
3	37	96.470	3	56	96.470
4	25	96.470	4	53	96.470
5	20	96.470	5	34	96.470
6	15	96.470	6	31	96.470
7	14	96.470	7	22	96.470
8	9	96.365	8	20	96.470
9	9	96.365	9	17	96.470
10	4	96.366	10	16	96.470
11	3	96.366	11	13	96.470
12	3	96.366	12	**8**	**96.470**
13	3	96.366	13	3	96.366
			14	3	96.366

For the Z_SCORE-based drug compounds regarding the GDSC2 dataset, the highest accuracy value with the minimum number of selected features is obtained with 11 selected features, assigning LASSO = 1 and mRMR = 2 rank-based weights, a total weight value of 6, and a 96.781% ACC (Table 8).

**Table 8:** The impact of the feature selection and weighting method in terms of the accuracy rate (%) for the Z_SCORE-based GDSC2 dataset.

LASSO = 1 and mRMR = 2			LASSO = 2 and mRMR = 1		
k	# of Features	RF	k	# of Features	RF
1	117	96.470	1	117	96.470
2	76	96.470	2	116	96.470
3	48	96.470	3	75	96.470
4	36	96.470	4	73	96.470
5	22	96.470	5	45	96.470
6	**11**	**96.781**	6	43	96.573
7	10	96.677	7	32	96.470
8	8	96.470	8	30	96.470
9	7	96.573	9	18	96.470
10	7	96.573	10	18	96.470
11	7	96.573	11	8	96.573
12	5	96.366	12	6	96.573
13	4	96.677	13	5	96.573
14	3	96.573	14	3	96.470

**Figure 2:**
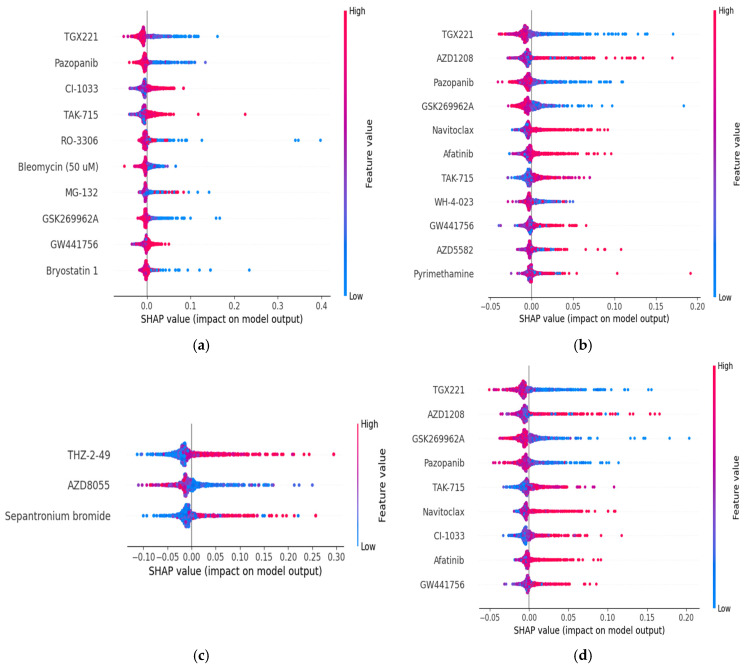
GDSC1 Dataset SHAP-based plots: (**a**)AUC; (**b**) LN_IC50; (**c**) RMSE; (**d**) Z_SCORE.

**Figure 3:**
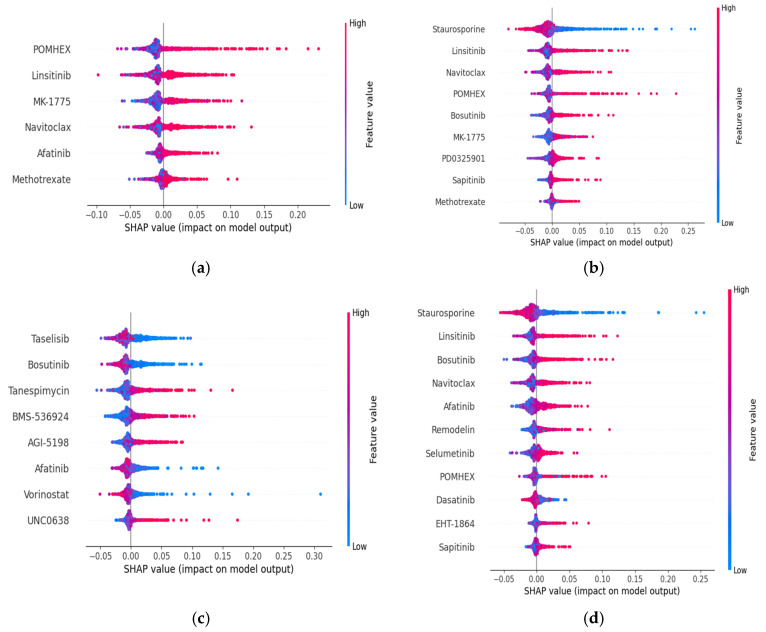
GDSC2 Dataset SHAP-based plots: (**a**)AUC; (**b**) LN_IC50; (**c**) RMSE; (**d**) Z_SCORE.

All selected feature names set for each specification are presented in Supplementary Table S1. We also constructed SHAP-based plots for each dataset and drug sensitivity metric by using the selected feature sets to observe the impact on the model output process (see Figure 2 and Figure 3). Red colors show higher values of a feature, and blue colors represent lower values of a feature. As can be observed from Figure 2, TGX221 is the most popular and significant (first-ranked) feature among them for the prediction model performance impact on the GDSC1 dataset. According to Figure 2c, THZ-2-49 is also the most efficient drug compound in terms of the RMSE metric on this dataset. We can say that a higher value of the TGX221 has a negative impact on the model output, while THZ-2-49 has a positive impact on the RF model output. TGX221 and THZ-2-49 drug compounds are the most critical compounds (i.e., ranked first) in terms of our feature selection methodology and SHAP-based results.

As can be observed from Figure 3, POMHEX is the most popular and significant (first-ranked) feature among them for the prediction model performance impact on the GDSC2 dataset according to AUC. Similarly, Staurosporine is the best compound in terms of LN_IC50, and Z_SCORE, and Taselisib is the most critical feature in terms of RMSE on the GDSC2 dataset. We can say that a higher value of Staurosporine has a negative impact on the model output, while POMHEX has a positive impact on the RF model output.

## 3. Discussion

In this study, we investigated the selection of drug compound features based on drug sensitivity metrics (i.e., LN_IC50, AUC, RMSE, and Z_SCORE) using the GDSC datasets. We aimed to identify discriminative and critical descriptors, drug compounds that are most predictive of response variability across GBM versus other cancer cell lines. This drug sensitivity-driven approach to the feature selection process is motivated by the increasing requirement for the machine learning model in precision oncology that is not only accurate but also interpretable and focused on actionable outcomes. Furthermore, to address the common issue of missing data in high-dimensional GDSC datasets, we employed iterative imputation as a data preprocessing step. Iterative imputation estimates missing values for each feature by iteratively using the relationships with all other available features. Compared to simpler imputation techniques like mean or median imputation, iterative imputation allows for more accurate and context-aware estimations. This approach is advantageous in complex biomedical datasets, where the relationships among variables are often nonlinear and interdependent. Iterative imputation has been shown to reduce bias and preserve the integrity of the original data distribution better than univariate methods by providing a convenient, flexible, and popular technique [35,36]. Despite its sophisticated characteristics, iterative imputation requires more computational resources compared to simpler methods [35,37]. We prioritized clear reporting of missingness and an iterative imputation strategy, rather than significance testing of differences that were widespread across the dataset. Since all variables exhibited missingness, we utilized a consistent iterative imputation strategy across features and experiments. This ensured dataset integrity and reduced bias that can arise from variable-specific handling. We analyzed the selected feature subsets for each dataset and metric with SHAP-based plots to observe and interpret their impact on the model prediction performance in terms of explainable AI. The results highlight the utilization of integrating drug response data directly into the feature selection process, potentially leading to more informative and biologically meaningful results. AUC and IC50 directly describe the sensitivity and potency of the drug, respectively, while the RMSE itself is not a sensitivity measure but rather a representation of the curve-fit error and the Z_SCORE is a means of data normalization. Given this, RMSE and Z_SCORE are less biologically interpretable but can provide a means to validate the method, as critical compounds may not be arguably selected by AUC and IC50, while RMSE and Z_SCOREs would not be used as ground truth labels of drug sensitivity.

One of the key findings of this work is that certain compound features consistently showed high importance with TGX221 emerging in GDSC1, while POMHEX emerged in GDSC2. TGX221 is not present in GDSC2, and POMHEX is not present in GDSC1. While compounds are shared between the datasets, 2 selected and shared compounds, namely Afatinib and Navitoclax, were identified, and some of the identified compounds above could not have been shared as they were not present in the opposite dataset. This illustrates that the data present in the datasets is critical to achieve drug repurposing. A combination of the datasets, however, would not be recommended given critical distinctions between the two sets. With harmonization, using dataset covariates allows the two datasets could be harmonized; however, only 122 compounds are shared between the two sets, hence limiting the data available to machine learning. We also found that the key identified compounds were critical to performance in AUC and IC50, but not in RMSE, given that it is not a sensitivity measure. TGX221 has a putative target PI3Kbeta and hence the PI3K/MTOR signaling pathway. TGX221 has been shown to induce apoptosis in GBM cells as well as impair migration and invasion [38]. POMHEX is listed as an unclassified compound in GDSC2. In the literature, POMHEX is described as a small molecule Enolase inhibitor that is aimed at targeting glycolysis in cancer and has been employed in ENO-1 deleted glioma cells [39]. Currently, neither compound has been employed in humans and there is no data beyond preclinical work.

There are several limitations that must be acknowledged. First, the GDSC datasets, while comprehensive, are still limited by the number of compounds tested and the cell line models used. The findings related to molecular and pharmacologic responses observed in vitro should not be interpreted as evidence of demographic effects in patient populations. The results may be shaped by how the cell lines were derived and the repository process selection used. Most of the drugs in these datasets are not FDA-approved. There are only 28 FDA-approved drugs for the GDSC1 and 31 FDA-approved drugs for the GDSC2 datasets among all drugs (Supplementary Table S4). Another limitation of this study is the lack of single-cell-level analysis. Because tumor samples exhibit substantial cellular heterogeneity, relying solely on bulk measurements can mask subpopulation-specific signals that are relevant to treatment response. As single-cell sequencing technologies continue to advance, capturing variability across cell states and cell types has become increasingly significant and critical for both mechanistic understanding and predictive modeling [40]. The lack of deep learning-based methods is a limitation of our study. As highlighted by Zhang et al. [41], deep learning techniques show considerable potential for improving predictive accuracy and clinical applicability. Despite this limitation, the framework delivers reliable and interpretable findings on pharmacologic responses, laying the groundwork for future deep learning applications. Incorporating such approaches will be an important direction for future research to further strengthen the translational impact of our framework. We employed stratified five-fold cross-validation to preserve the natural class distribution in each fold. This approach ensures that both training and evaluation sets reflect the true imbalance of the dataset. This process prevents minority classes from being excluded during the training or evaluation phase. No oversampling or undersampling techniques were applied, as our goal was to maintain biological validity and preserve robustness for the results and rather than artificially balance the datasets. While the stratified cross-validation approach preserved class proportions across folds, it did not resolve the underlying imbalance problem. While overall accuracy was around 96%, the balanced accuracy was around 55%, underscoring the challenge of minority-class detection. We report this metric transparently and note that our study’s primary contribution lies in the biological insights from cell line drug responses by employing the feature selection methodology. In summary, selecting drug compound features based on sensitivity data shows strong potential for improving how we model and understand drug responses in GBM versus other cancer cell lines. This approach improves predictive model performance, and it can also offer valuable insights. Since both data and computational tools continue to evolve, we believe this type of hybrid feature selection will play an important role in advancing precision oncology and supporting more effective drug discovery.

## 4. Materials and Methods

This section outlines the experimental design, data sources, and analytical methods used to investigate our feature selection and weighting methodology, including definitions, techniques, and prediction models utilized in this study.

### 4.1. Datasets

The Genomics of Drug Sensitivity in Cancer (GDSC) [42] Project is a Wellcome-funded collaborative effort between the Cancer Genome Project at the Wellcome Sanger Institute (UK) and the Center for Molecular Therapeutics at Massachusetts General Hospital Cancer Center (USA). This partnership brings together the strengths of both institutions to identify cancer biomarkers that can help predict which genetically defined patient subgroups are most likely to benefit from specific cancer treatments [42].

The characteristics of the utilized GDSC datasets (i.e., GDSC1 and GDSC2) are presented in Table 9 in detail. The GDSC1 and GDSC2 datasets are two complementary parts of the GDSC project, created to systematically profile the sensitivity of cancer cell lines to anti-cancer drugs and associate this with genomic features. Differences between the datasets include the time period when the data was obtained, with GDSC2 being more recent, the dose design used for different drug concentrations to measure dose–response relationships, the control and storage of the compounds employed, and in common they have the analytic approach based on fitting a dose–response curve and extracting summary metrics such as IC50 (i.e., half-maximal inhibitory concentration) and AUC (Area Under the Curve). Given the above, they are treated as separate analyses but are complementary to each other [42]. There are 122 shared drug compounds (i.e., features) between the GDSC1 and GDSC2 datasets (Supplementary Table S2). Additionally, 22 of these shared drug compounds are in our selected feature set in terms of using all metrics and datasets (Supplementary Table S3). We used the same parameter settings for both datasets in terms of clinical usability or drug repurposing.

### 4.2. Methodology

This section provides a general overview of the feature selection and weighting architecture we employ, including a brief description of the essential methodologies.

#### Proposed Scheme

**Figure 4:**
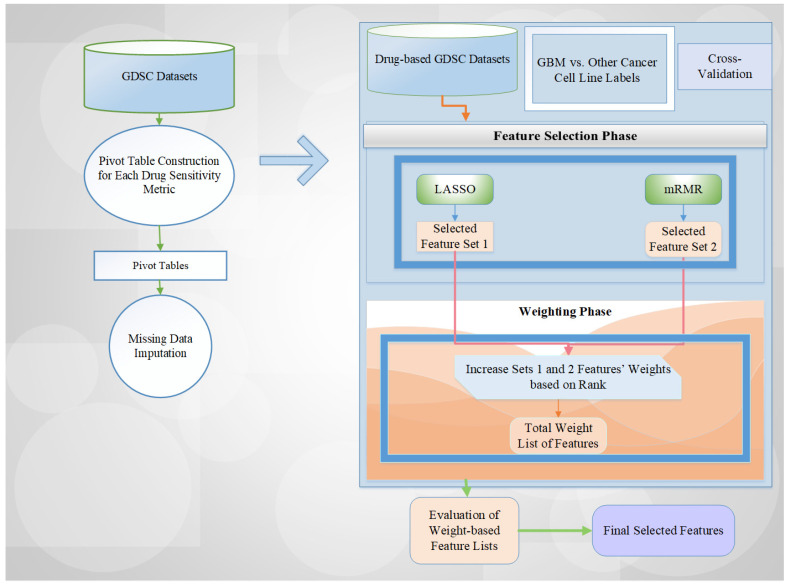
The overview of our proposed scheme for feature selection tasks on drug compound-based GDSC datasets.

This study adopted our previous similar studies from Tasci et al. [7,43] in terms of the methodology used. We constructed drug compound-based cell line datasets with GBM/other cancers (i.e., binary) class labels. Afterwards, our previous methodology was applied to the related drug compound-based GDSC datasets for each drug sensitivity metric. Our utilized scheme includes two phases: (i) Feature weighting; (ii) Feature selection (Figure 4).

The general operation of the methodology can be summarized as follows: Two popular feature selection methods, LASSO and mRMR, are utilized for the feature selection tasks. Then, we keep all selected features for each fold of the cross-validation in a list with a count. We increase the count of the selected features with respect to feature weights (i.e., 1 or 2 depending on their importance). Furthermore, we evaluate the total value of the feature weights for the different combination schemes, such as the total weight value in terms of accuracy rate. After trying all combinations among these schemes, we select the final feature set that has the maximum accuracy rate with the minimum number of selected features. This approach provides an efficient way to reduce dimensionality for high-dimensional datasets by determining relevant feature names.

In other words, we employed a rank-based feature weighting methodology in this study. LASSO and mRMR feature selection methods were assessed according to their predictive performance (i.e., accuracy for classification tasks), and the better-performing method was assigned a higher weight value. For each cross-validation fold, features selected by LASSO or mRMR received weights of 2 or 1, respectively, contributing to their cumulative importance scores. In this study, k (i.e., weight value) values between 1 and 15 were investigated due to the five-fold cross-validation. If the five-fold cross-validation is used and a feature is chosen in each fold by both feature selection methods, the total maximum weight value will be 15 (i.e., 2 × 5 folds + 1 × 5 folds). For example, if there is no selected feature chosen in each fold by both feature selection methods, the maximum total weight value will be lower than 15. If a feature is selected in only one fold of cross-validation by only one feature selection method, the minimum total weight will be 1. Then, we evaluated all ranked weight combinations to identify the most effective selection scheme and to optimize predictive performance for the corresponding dataset (i.e., maximum accuracy rate with minimum number of selected features).

## 5. Conclusions

This study presents a hybrid machine learning-based feature selection approach for drug sensitivity-driven compound selection in cancer cell lines. Random forest machine learning model with different rank-based weighting schemes is assessed to provide robust drug compound candidates for discriminating GBM and other cancer lines with discriminative features from a high-dimensional/large-scale bioinformatics dataset. Advancements and ongoing research in this field are set to substantially improve GBM treatment outcomes while making significant contributions to cancer-related work, particularly in drug discovery and personalized medicine. Future work can focus on applying our hybrid feature selection method across diverse biomedical datasets to further improve the generalization of the model and results. Future efforts can also aim to incorporate single-cell-derived features into our framework, drawing on methodologies such as those presented by Lai et al. [40], which utilize single-cell-resolved signatures to improve response prediction and deepen biological interpretation. As another direction of future work can combine cell line-based results with genome-wide association studies-derived cohorts to better connect molecular mechanisms and enrich this study scope. We will also focus on expanding datasets to improve minority-class representation in a biologically consistent manner.

## Figures and Tables

**Table 9:** GDSC utilized the dataset characteristics and methodology.

	GDSC1 Dataset	GDSC2 Dataset
Total # of Instances	968	963
# of GBM/# of Non-GBM	33/935	34/929
Total # of Features	378	286
Feature Selection Methods	mRMR + LASSO	
Cross-Validation Type	Stratified 5-fold CV	
Classification Model	Random Forest	

## Data Availability

The data used in this study are available in Genomics of Drug Sensitivity in Cancer (GDSC) at https://www.cancerrxgene.org/downloads/bulk_download, accessed on 5 September 2025. The related datasets were derived from this resource available in the public domain.

## Supplementary Materials

The following supporting information can be downloaded at: https://www.mdpi.com/article/10.3390/ijms262411871/s1.

## Abbreviations

ACC	Accuracy
AI	Artificial Intelligence
AUC	Area Under the Curve
CRT	Chemoirradiation
FDA	Food and Drug Administration
FS	Feature Selection
FW	Feature Weighting
GBM	Glioblastoma Multiforme
GDSC	Genomics of Drug Sensitivity in Cancer
LASSO	Least Absolute Shrinkage and Selection Operator
LN_IC50	Half Maximal Inhibitory Concentration
ML	Machine Learning
MRMR	Minimum Redundancy–Maximum Relevance
NCI	National Cancer Institute
NIH	National Institutes of Health
RF	Random Forest
RMSE	Root Mean Squared Error
RT	Radiation Therapy
SHAP	SHapley Additive exPlanations
TMZ	Temozolomide
Z_SCORE	Standard score
